# *“‘You lose your hair, what’s the big deal?’ I was so embarrassed, I was so self-conscious, I was so depressed:”* a qualitative interview study to understand the psychosocial burden of alopecia areata

**DOI:** 10.1186/s41687-020-00240-7

**Published:** 2020-09-11

**Authors:** Natalie V. J. Aldhouse, Helen Kitchen, Sarah Knight, Jake Macey, Fabio P. Nunes, Yves Dutronc, Natasha Mesinkovska, Justin M. Ko, Brett A. King, Kathleen W. Wyrwich

**Affiliations:** 1 Clinical Outcomes Assessment, DRG Abacus (part of Clarivate), The Lexicon, Mount Street, Manchester, M2 5NT UK; 2 Lilly Bio-Medicines, Eli Lilly and Company, Eli Lilly and Company, Lilly Corporate Center, Indianapolis, IN 46285 USA; 3 University of California Irvine Dermatology Clinical Research Center, Hewitt Hall Building, 843 Health Sciences Road, Room 1001, Irvine, CA 92697 USA; 4 Stanford Dermatology, Stanford University School of Medicine, 291 Campus Drive, Li Ka Shing Building, Stanford, CA 94305-5101 USA; 5 Department of Dermatology, Yale School of Medicine, 333 Cedar Street, LMP 5040, New Haven, CT 06520 USA; 6 Patient-Focused Outcomes Center of Expertise, Eli Lilly and Company, Lilly Corporate Center, Indianapolis, IN 46285 USA

**Keywords:** Alopecia areata, Quality of life, Psychosocial, Qualitative, Health-related quality of life, Conceptual model

## Abstract

**Background:**

Alopecia areata (AA) is characterized by hair loss that can affect the scalp and body. This study describes the psychosocial burden of AA.

**Methods:**

Participants diagnosed with AA who had experienced ≥50% scalp hair loss according to the Severity of Alopecia Tool (SALT) were identified by clinicians. A semi-structured interview guide, developed with expert clinician input, included open-ended questions to explore patients’ experiences of living with AA. Data were thematically analyzed to identify concepts and relationships.

**Results:**

Participants (*n* = 45, 58% female, mean age 33.3 years [range 15–72], mean SALT 67.2 [range 0–100]) described the AA diagnosis as “devastating”. Both males and females reported emotional and psychological impacts of AA including feeling sad/depressed (*n* = 21), embarrassed/ashamed (*n* = 10) and angry/frustrated (*n* = 3). Patients felt helpless (*n* = 5) due to the unpredictability of disease recurrence, and anxious (*n* = 19) about judgement from others. Many patients avoided social situations (*n* = 18), which impaired relationships and increased isolation. Coping strategies included concealment of hair loss through wigs or make-up, although fear of the displacement of these coverings also caused anxiety and the avoidance of activities that could result in scalp exposure (*n* = 22). Some patients became more accepting of AA over time, which lessened the emotional impact, though efficacious treatment was still desired. A conceptual framework was developed, and a conceptual model was created to depict the relationship between the physical signs/symptoms and the associated psychosocial effects of AA.

**Conclusion:**

AA impairs patients’ emotional and psychological wellbeing, relationships and lifestyles. Greater disease awareness and effective treatments are needed.

## Background

Alopecia areata (AA) is a chronic autoimmune inflammatory disease characterized by hair loss that can affect the scalp, face (e.g. eyebrows and eyelashes) and body [1]. A recent (2017) national US population survey estimated lifetime prevalence of AA at approximately 2.5% [2], slightly higher than previous regional estimates of 1.7% (1970–1989) [3] and 2.1% (1990–2009) [4]; although current prevalence was found to be similar to upper estimates from the 1970s at around 0.2% (0.09% moderate–severe disease) [2]. AA can occur at any age and affects both children and adults; median age at diagnosis is 33 years [4], and diagnosis is more likely during childhood for males and during adolescence for females [5]. There is no preventative therapy or currently approved therapy that sustains remission [6].

The impact of AA on patients’ health-related quality of life (HRQoL) has been explored in several quantitative studies that have identified low HRQoL in adults [7–13], in children [14, 15], and in family members (of those affected by AA) [8, 15, 16], with particularly significant impairment in emotional functioning and mental health [17]. These studies involved quantitative collection of patient-reported data and did not seek to qualitatively conceptualize patients’ disease experience. The extent of HRQoL impairment in patients with AA appears to be similar to patients with other chronic skin diseases including atopic dermatitis and psoriasis [8], although the sensitivity of these findings may be limited due to the absence of an AA-specific measure of HRQoL.

In qualitative explorations, AA has been observed to impact patients’ confidence, self-esteem, and socialization [18, 19], with some researchers postulating greater impact on females than males [19–21]. Due to the negative cultural connotations associated with hair loss, people with AA often adopt coping strategies [22], including wig use [18, 21–23] and social avoidance [18, 23]. Access to formal psychological support can be limited [22], but nonetheless, persons with AA may become more accepting of their condition over time [23].

Few qualitative studies have conceptualized the relationship between AA signs/symptoms and the psychosocial burden of this medical disease [24], and while conceptual frameworks of the physical and psychosocial burden of skin disease have been hypothesized by Chren in the development of the Skindex [25, 26], no conceptual model to represent the particular effects of AA has been developed to inform patient-reported outcome (PRO) measurement.

This study qualitatively explored the symptom experience and psychosocial burden of living with AA. Additionally, through the thematic analyses of the study data, we developed a conceptual model to depict the patient experience of AA that can be used to underpin future disease-specific assessments of the impact of AA.

## Methods

### Ethics

This qualitative study was designed to elicit the patient experience of AA and to develop and cognitively test PRO measures for key physical AA signs/symptoms (results of this latter objective are reported elsewhere [27, 28]). The study protocol was reviewed and approved by Western Institutional Review Board (ref #20171820).

### Sample

This study included adults or adolescents (aged ≥12 years) with AA who had experience of hair loss involving 50% or more of their scalp, as assessed by the Severity of Alopecia Tool (SALT) (Table 1). Adults were the primary population of interest; a small sample of adolescent patients were included to gain insight into their experience and inform future research. Some of the recruited subjects had experience of eyebrow and/or eyelash involvement in order to allow exploration of these signs/symptoms and their impacts. Interviews were conducted in two rounds; interim analyses were completed after 30 interviews (Round 1). Following the first 30 interviews, eligibility criteria were modified to focus on the experiences of adults with severe hair loss (SALT ≥50%) at the time of recruitment in Round 2.

**Table 1:** Eligibility criteria

	Round 1 (*n* = 30)	Round 2 (*n* = 15)
**Inclusion criteria**	• Aged ≥12 years at time of consent • EITHER: ◦ Severe AA diagnosed by a clinician and determined by SALT ≥50% and never treated with JAKi, **or** ◦ Has received successful JAKi treatment, with SALT ≥50 prior to treatment	• Aged 18–60 years at time of consent • Severe AA diagnosed by a clinician and determined by SALT ≥50% • Current AA episode lasting > 6 months and no scalp hair regrowth over the past 6 months
	• A history of AA episodes lasting > 6 months but < 8 years • Sufficient physical, cognitive, reading and linguistic capacities to allow patients to actively participate in a 90 − minute interview • Fluent in English and can give documented informed consent	
**Exclusion criteria**	• Diagnosed with androgenic alopecia	• Diagnosed with other forms of alopecia including male or female pattern hair loss • Has experienced ≥95% scalp hair loss for ≥8 years
	• Diagnosed with active psoriasis, atopic dermatitis, or other dermatological condition that could be severe enough to impact this study, or any other serious condition that could interfere with this study • Known substance abusers	

*Abbreviations: AA* Alopecia areata, *JAKi* Janus kinase inhibitors, *SALT* Severity of Alopecia Tool

### Interview guide

A semi-structured interview guide was developed for each of the two study rounds; both of which included open-ended questions to explore patients’ experience of AA: *‘Tell me about your experience living with alopecia?’* Round 1 interviews also included specific psychosocial follow-up probes: *‘How does having alopecia make you feel?’* and explored the impact of AA on aspects of daily life, school/work, daily activities, and relationships: *‘Tell me how your alopecia affects your daily activities, if at all?’* and *‘Tell me how your alopecia affects you at school/work if at all?’.* During the Round 2 interviews, experience of AA was explored with open-ended questions but probed in less detail. Questions to cognitively test several PRO measures were also included in both rounds; these findings are reported elsewhere [27, 28].

### Recruitment procedure

Referring clinicians at the University of California – Irvine, Yale University, Northwest Dermatology Research Center in the US, and SKiN Centre for Dermatology in Canada described the study to potential participants and, for those aged < 18 years, their parent/guardian. Interested individuals (and their parent/legal guardian) were then provided with an information and consent form (ICF) that further described the study. Adult participants signed the ICF prior to data collection. Adolescent participants (aged 12–17 years) and their parent/guardian both signed the ICF.

### Interview procedure

Participants attended a 90-min, one-to-one, face-to-face interview between October 2017 and March 2018 at the referring clinic or a nearby meeting room. Interviews were conducted by an experienced qualitative interviewer trained in concept elicitation techniques. Interviews were audio-recorded and transcribed verbatim. Patients received an honorarium of $150 USD/CAD for their participation in a 90-min, face-to-face interview.

### Analysis

Transcripts were reviewed and all identifying information were removed. Participants were allocated codes to anonymize reporting which included the chronological interview order and their gender. For example, participant 33-F was interviewed 33rd and was female.

Transcripts were coded using ATLAS.ti Version 7.5. Thematic analysis [29] took a phenomenological approach, seeking to understand the realities of participants experiences [30]. Descriptive codes were assigned to quotes within each transcript; in this way concepts and themes were identified. The first two transcripts informed a preliminary codebook, which was applied to the remaining interview transcripts. When new codes emerged, earlier transcripts were reviewed to ensure that the new concept was not overlooked previously. Coding was led by two analysts in constant communication. The study investigator monitored and resolved coding and final analysis. Code-quotation outputs were checked during analysis and reporting and updated if any coding errors were found; where more than one interpretation could exist, this was discussed with the original coder.

#### Conceptual saturation

Conceptual saturation, the point at which no new concept-relevant information emerges [31], was assessed in line with industry guidelines [32, 33] to guide sampling and analysis. A target sample size of *N* = 30 was hypothesized as initially sufficient to explore conceptual saturation [33–35].

#### Theoretical framework and conceptual model

A theoretical framework of the effects of AA was developed, informed by the frameworks previously developed by Chren in the development of the Skindex [25, 26]. This framework hypothesized that skin diseases affect patients in both psychosocial and physical ways [26], with psychosocial effects comprising emotional and functional effects [25]. From this framework, a conceptual model, hypothesizing the relationships between AA signs/symptoms and impacts was developed [25].

## Results

### Sample

A total of 45 patients, aged 15–72 years and with a near equal split of females and males (58% and 42% respectively), participated in the study (Table 2). Patients had been diagnosed with AA for 11.8 years on average and over half (*n* = 23, 51%) were not receiving treatment. Thirty-three patients (73%) had ≥50% scalp hair loss at the time of recruitment.

**Table 2:** Sample characteristics

Sample characteristic	N (%)(N = 45)
Years since diagnosis, mean [range]	11.8 [1–47]
SALT score^a^, mean [range]	67.2 [0–100]
Current treatment, n (%)^b^	
JAKi	15 (33)
Other treatment(s)	7 (16)
Unknown, either JAKi or placebo^c^	2 (4)
No treatment	23 (51)
Gender, n (%)	
Male	19 (42)
Female	26 (58)
Age, mean [range]	33.3 [15–72]
Ethnicity/race, n (%)	
Asian	9 (20)
Black or African American	2 (4)
White	25 (56)
Hawaiian or Pacific Islander	1 (2)
Hispanic	5 (11)
Other	3 (7)
Country, n (%)	
United States	42 (93)
Canada	3 (7)
Education, highest certificate achieved	
No high school diploma	9 (20)
High school diploma or equivalent	15 (33)
Associate’s degree	2 (4)
Bachelor’s degree or higher	19 (42)

*Abbreviations: JAKi* Janus kinase inhibitors, for example tofacitinib, *SALT* Severity of Alopecia Tool

^a^SALT scores were clinician-reported and were calculated within a mean 0.6 months of the interview (range 0–7 months)

^b^Non-mutually exclusive. Other treatments include Biotin Forte with zinc (n = 1), Clobetasol 0.05% ointment (n = 2), Diphenylcyclopropenone (n = 2), Excimer (*n* = 1), Intralesional Kenalog (n = 3), Luxiq foam (n = 1), Rogaine (n = 1), Slow-release iron (n = 1), Vitamin E (n = 1)

^c^Two patients were in a clinical trial and it was unknown if they were receiving JAKi or placebo

Note: due to rounding some percentages may not total 100%

### Saturation analysis

Saturation analysis revealed that all concepts identified were spontaneously reported by patients in the first 24 interviews and that a comprehensive understanding of the patient experience of AA was obtained during the first 30 interviews (Wyrwich, K. W. et al.: The role of patients in alopecia areata endpoint development: Understanding physical signs and symptoms. Forthcoming.). Subsequent interviews were conducted with the primary purpose of debriefing PRO measures; nonetheless, limited concept elicitation data were collected, analyzed, and compared with the saturation grids. No new concepts emerged in the final 15 interviews.

### Theoretical framework and conceptual model of the effects of alopecia areata

**Fig. 1:**
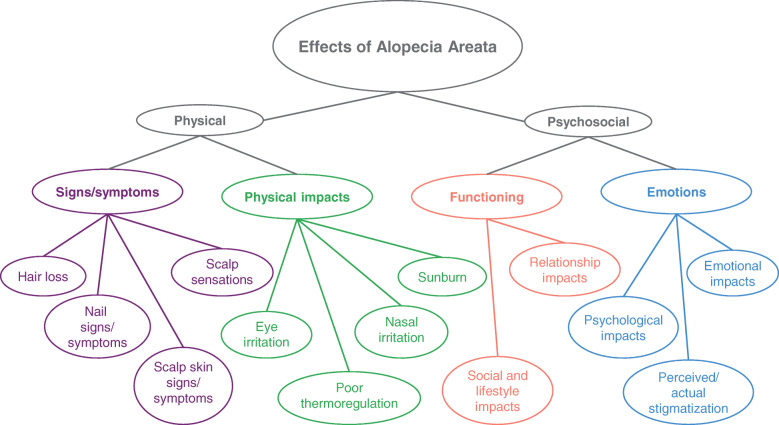
Conceptual framework of the effects of alopecia areata

**Fig. 2:**
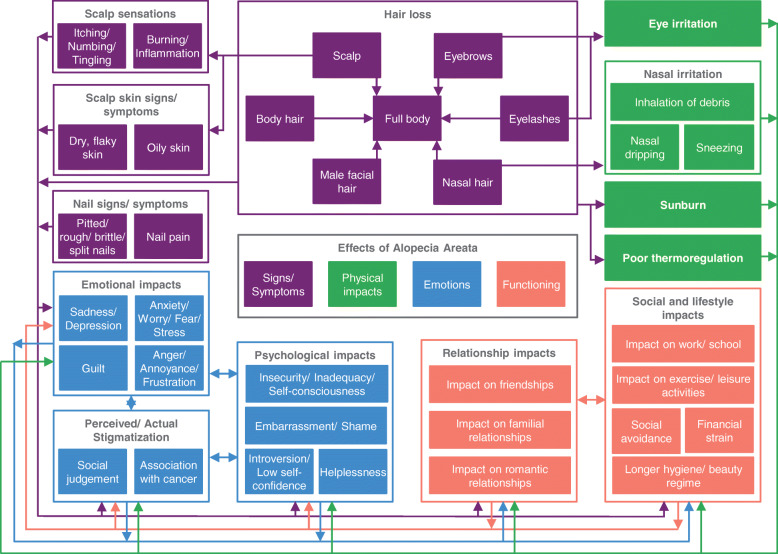
Conceptual model of the effects of alopecia areata

A total of 37 concepts emerged from the data. Following Chren’s frameworks [25, 26], these concepts were first grouped into either a physical or a psychosocial domain with concepts placed in the latter further separated into emotion concepts and functioning concepts. An additional sub-domain emerged; it was identified that physical effects could be grouped into primary signs/symptoms and secondary physical impacts of those signs/symptoms, such as eye irritation resulting from loss of eyebrows and/or eyelashes, to create the conceptual framework of the effects of alopecia areata (Fig. 1). Within each sub-domain, related concepts were grouped together, and relationships between concepts and sub-domains were posited using the detailed interview data to develop a conceptual model of the effects of AA (Fig. 2).

### Signs/symptoms and physical impacts of AA

All participants had experienced hair loss, the primary sign/symptom of AA. However, this was experienced to varying extents, from scalp hair loss alone (*n* = 6, 13%) to complete hair loss of the entire body (*n* = 28, 62%). Other symptoms included scalp sensations (*n* = 12, 27%) and nail involvement (*n* = 14, 31%). Secondary to these signs/symptoms, participants had experienced a number of physical impacts of AA, including eye (*n* = 22, 49%) and nose (*n* = 8, 18%) irritation, poor thermoregulation (*n* = 3, 7%), and sunburn (n = 3, 7%) (Table 3).

**Table 3:** Signs/symptoms and physical impacts of AA

Concept	Patients with experience of concept, n (%) (*N* = 45)	Example quote(s)
Hair loss	45 (100)	
Scalp	45 (100)	− *“It started off as spots. Then the spots grew bigger and bigger, and at times it regrows a bit. And, again, it’s kind of an episode. You won’t lose hair all the time, but, when you do lose hair, it’s kind of drastic to see.” (35-M)*
Eyebrows	38 (84)	− *“They’re pretty sparse. There’s probably like maybe five [hairs] in each eyebrow.” (19-F)*
Eyelashes	32 (71)	− *“It started on one eye and just kind of progressively like went down the lid, falling out. The other one started falling and eventually all the eyelashes were gone.” (11-M)*
Nasal hair	14 (31)	− *“I lost the hair in my nose as well, so, yes, again, you don’t get the protection again there.” (25-F)*
Male facial hair	11 (58^a^)	− *“I had a full beard, literally. A full beard and it’s all gone.” (26-M)*
Body hair	36 (80)	− *“I’ve lost all the hair on my arms and on my legs. Started losing the hair underneath my, on my armpit region.” (08-M)*
Full body	28 (62)	− *“Well, I have no hair anywhere at all. So it’s affected from my head to my legs, my under arms, private areas, everywhere.” (41-F)*
Nail signs/ symptoms	14 (31)	
Pitted/ Rough/ Brittle/ Split	14 (31)	− *“They’re very weak and they’re bumpy so when I apply nail polish there’s like ridges, waves. It’s just superficial but I like to have my nails look nice.” (06-F)*
Nail pain	2 (4)	− *“Since the nail is not attached all the way […] if I were to whack it, be clumsy or whatever, I’d be in pain for days.” (24-F)*
Scalp skin signs/ symptoms	12 (27)	
Itching/ Numbing/ Tingling	10 (22)	− *“When hair was growing it was very itchy.” (29-F)* − *“I kind of feel like a light, numbing feel. There’s a difference in the texture of the way my skin feels when it gets touched or rubbed or hit. So I actually do feel the difference. If I were to, say, touch my arm, I can feel that more than my scalp.” (01-M)*
Burning/ Inflammation	4 (9)	− *“Sometimes I get red bumps and a lot of times the area, it will get red and hot, you can feel it before the hair starts to come out.” (18-F)*
Dry, flaky skin	4 (9)	− *“I noticed it was very dry and flaky.” (05-F)*
Oily	1 (2)	− *“I guess the hair soaked up usually sometimes the oils, natural oils.” (07-M)*
Eye irritation	22 (49)	− *“When I started losing my eyelashes and they’ve kind of come and gone, I noticed me eyes are much more irritated now. Like in the last couple of years, my eyes will get red, they burn, I feel like there’s stuff in my eyes.” (08-M)*
Nasal irritation	8 (18)	
Inhalation of debris	3 (7)	− *“I breathe in more than your average person that has hair. I guess it’s like a filter. I notice a big difference. I got to blow my nose out every night. I got to shower at least 20 min in steam to get it all out.” (01-M)*
Nasal dripping	3 (7)	− *“I feel like I have constant nasal drippage, and it’s disgusting […] It’ll just like start, liquid will start pouring out.” (27-F)*
Sneezing	2 (4)	− *“When I sneeze I do it either five or six times in a row […] I didn’t have that when I had nose hairs.” (07-M)*
Poor thermoregulation	3 (7)	− *“During sleep, during waking hours, from the cold weather to the hot weather, you can’t regulate your body temperature.” (24-F)*
Sunburn	3 (7)	− *“Your hair gets really thin so sun passes through it. So I would get sunburns on my scalp very easily, I would feel my head burning.” (10-F)*

^a^Calculated as a percentage of male patients (*N* = 19)

### Psychosocial impact of AA

The number of patients discussing their experience with each psychosocial concept included in the conceptual model is presented in Table 4. Participants were not directly questioned regarding each of these concepts, and discussion of psychosocial impacts was limited during the Round 2 interviews; thus, these numbers are likely under-representative.

**Table 4:** Number of patients discussing their experience with each psychosocial concept included in the conceptual model

Concept	Patients discussing experience of concept, n (%) (*N* = 45)
Perceived/ Actual stigmatization	
Social Judgement	20 (44)
Association with cancer	12 (27)
Relationship impacts	
Impact on romantic relationships	8 (18)
Impact on familial relationships	10 (22)
Impact on friendships	16 (36)
Emotional impacts	
Sadness/ Depression	21 (47)
Anxiety/ Worry/ Fear/ Stress	19 (42)
Guilt	3 (7)
Anger/ Frustration	3 (7)
Embarrassment/ Shame	10 (22)
Psychological impacts	
Insecurity/ Inadequacy/ Self-consciousness	25 (56)
Helplessness	5 (11)
Low self-confidence/ Introversion	9 (20)
Social and lifestyle impacts	
Impact on exercise/ leisure activities	22 (49)
Financial strain	4 (9)
Longer hygiene/ beauty regime	15 (33)
Social avoidance	18 (40)
Impact on work/ school	18 (40)

Hair loss in areas that were visible to others were most psychosocially burdensome to patients. Most (*n* = 35, 78%) patients reported that scalp hair loss was their most bothersome symptom because it was most noticeable. Patients who reported eyebrow loss as most bothersome (*n* = 7, 16%) were male patients who mostly felt eyebrow loss was less socially accepted than male scalp baldness. One (2%) patient had a personal preference for *“nice eyelashes than nice [scalp] hair”* (17-M). Other individuals were most bothered by the functional impacts of nose hair and body hair loss (each *n* = 1, 2%), which resulted in nasal dripping and poor thermoregulation/discomfort, respectively.

-
  *“I think when you first see someone it’s their hair, at least for me, like the hair is everything.” (34-F)*
-
  *“[Eyebrows] gives you more of a facial recognition, you know, I feel more as an outcast without my eyebrows. Just it seems different. People look at you like, oh, what’s going on with him, he doesn’t have his eyebrows.” (40-M)*

#### Emotions

Almost all patients described an emotional impact of living with AA; for many patients, this was the most significant aspect of the condition. Patients described feeling sad or depressed about their AA diagnosis (n = 21, 47%), a feeling that, for some, lasted indefinitely. The lack of treatment options available for AA caused patients to feel helpless (*n* = 5, 11%), unable to control the progression of the condition, and patients were anxious, worried, fearful, and/or stressed about disease recurrence or worsening:

-
  *“It was devastating when it first started. And when I first lost it all four years ago, I cried a lot. [ …] And it took me about two years. I really – I really had to mourn the loss of my hair.” (05-F)*
-
  *“It made me pretty sad for a long amount of time.” (14-M).*
-
  *“It makes you feel like out of control, [ …] I was a young professional and I was very successful at work, but yet I felt like a failure all the time because I couldn’t control this thing.” (30-F)*
-
  *“The uncertainty of not knowing which way it was going to go and why and anticipating that I was going to be completely bald was the most horrific part of the whole experience.” (24-F)*

Patients reported feelings of insecurity, inadequacy, and/or self-consciousness (*n* = 25, 56%) due to the changes in their appearance, which for some had impacts on their identity. Some females reported feeling inadequate as women, equating their hair loss to a loss of femininity. Body hair loss appeared to be less problematic for patients than hair loss on the scalp and face because it was less visible to others, however, one male described feeling less masculine without leg hair.

-
  *“I was so embarrassed, I was so self-conscious, I was so depressed about it. I concealed it, concealed it, concealed it.” (24-F)*
-
  *“The most important thing that affected me was how I felt about myself. [ …] I felt like I was not, like, worth loving.” (29-F)*
-
  *“When I go to a room with all these other women, I feel inferior because I have no eyelashes.” (27-F)*
-
  *“Looking in the mirror is emotionally taxing in a way that I feel like it probably shouldn’t be.” (28-M)*

The emotional experience of AA could change over time for some patients. While diagnosis or the initial stages of dealing with AA were described as ‘traumatizing,’ ‘devastating,’ or ‘terrifying,’ some patients described how they had learned to accept their hair loss or their appearance, either partially or fully. Some patients described how they turned their diagnosis into a positive and embraced their individuality:

-
  *“My first year was more tough than it is now. [ …] I’ve accepted it. It’s over. It is what it is. I can’t change it. Until you get to that point, it’s very difficult.” (26-M)*
-
  *“Overall, I feel like it’s kind of molded me into being somebody who strives to be kind of unique, [ …] who likes kind of not exactly fitting in with the crowd necessarily, to some degree. [ …] it’s made me more confident with just who I am, like I am this person, and I’m okay with it.” (03-M)*

Others, however, found it enduringly difficult to cope with the condition. Some patients reported feeling angry or frustrated (*n* = 3, 7%), particularly at the unavailability of treatments and the lack of public understanding about the condition. The extent of this emotional reaction caused some patients to feel guilty (n = 3, 7%) and question the acceptability of their own negative feelings caused by AA:

-
  *“Like people really don’t understand how hard it is. It’s only hair, but it really messes up everything in your life.” (25-F)*
-
  *“I don’t want to say I lost it, but like I was not good with it. I was not happy about it. It did upset me a lot.” (28-M)*
-
  *“I feel guilty because I see people that have severe disabilities and it could be so much worse for me and then I’m like I can’t believe I’m so upset about hair. But at the same time, I can’t help it.” (09-F)*

Many patients (*n* = 20, 44%) described situations when they had perceived or experienced negative judgement from others, which could cause them to feel embarrassed or ashamed (*n* = 10, 22%), and anxious, worried, fearful, and/or stressed about social situations. Patients worried that others may notice their hair loss, and described how this made them less confident in their appearance (*n* = 9, 20%). For some patients, this affected their personality, causing them to become more introverted:

-
  *“People never really look at me like a full human being sometimes. They always look at me like in a weird way, like, ‘he doesn’t have eyelashes and eyebrows, and he doesn’t have hair.’” (04-M)*
-
  *“Does it affect me physically? No. The ability to move around? No. To be around people? Yeah, it does. It does play a big role.” (01-M)*
-
  *“I was okay being bald, but other people around me made it really difficult... [ …] just dealing with the public and the morons, and, you know, and the jerks.” (09-F)*

Twelve (27%) patients described how other people sometimes assumed that they had cancer due to scalp hair loss being associated with chemotherapy. This led some to feel that they were unnecessarily treated with fear, sympathy, or caution:

-
  *“[My supervisor] noticed that I was wearing a wig, and she thought I had like some kind of chemo [ …]. I mean like it all came from good intentions. She wanted to care for me but then it’s like ‘oh yeah I didn’t have chemo,’ and I didn’t want to be looked as a person who was really sick.” (29-F)*

#### Functioning

Many patients concealed their AA using wigs, make-up, and cosmetic procedures such as microblading, and some (both males and females) shaved their head entirely. Patients described how their daily hygiene or beauty routines centered on hiding their hair loss, which took time (*n* = 15, 33%), and affected them financially (*n* = 4, 9%):

-
  *“If I’m going somewhere, then I have to think about enough time to put on eyebrows, put on the eyeliner, am I going to wear a wig, am I going to wear a hat, am I going to wear a scarf, am I going to go bald? You know, so I have to kind of think about those things.” (05-F)*

Exercise and physical activities, in particular swimming, were impacted by AA due to fears that their AA would be noticed by others due to, for example, wig or makeup displacement. Many patients (*n* = 22, 49%) therefore avoided these activities:

-
  *“I felt like I lost so much with my kids growing up at the beginning, because I wasn’t able to be there for them. Like I’ve never been inside the pool with them. I was always afraid of the wind blowing and everyone noticing that it was a wig.” (25-F)*
-
  *“I don’t even know how I’d do if I didn’t have to constantly [be] thinking about, [ …] ‘can I [do that activity] with my wig?’” (06-F)*

Patients described how the emotional impacts of AA, such as lowered confidence and self-consciousness, affected their performance at work and at school (*n* = 18, 40%). One participant altered their working hours to avoid interacting with others, and another did not work at all due to their AA. Two participants perceived they had lost out on jobs due to judgement from potential employers who believed them to be severely ill:

-
  *“A lot of times I was presenting things to boards or to staff or to other people in my firm or whatever, and it just always, you know. I think that once it started getting worse, I had a lot less self-confidence about everything I did.” (30-F)*
  *“I’m a cashier. So now my eyebrows are gone, I put my head down low, and then you can’t see my eyes, [ …]. I don’t really give that customer service like I should … so that’s really affected me.” (36-M)*

Social impacts were considerable. Patients sometimes avoided attending social events and/or mixing with other people, particularly during their school years where the heightened social pressure (and recent prominence of social media) made them fearful of standing out (n = 18, 40%):

-
  *“Once the alopecia was at its worst point that I had, I was just like, a homebody, you could say. I wouldn’t want to go out. [ …] I would avoid it. I wouldn’t go to, like, parties where you have to suit up. Yeah, I missed, like, my friend’s wedding.” (31-M)*
-
  *“In terms of social media, I guess I take less pictures. That type of thing. [ …] You see like judgment completely passed on Facebook where you see what happened to you. ‘Are you dying? Do you have cancer?’ [ …] I’ve tended to stay away from it just because I don’t want to be involved in it.” (26-M)*

Participants described impacts (both positive and negative) of AA on their relationships with their partners (*n* = 8, 18%), family (*n* = 10, 22%), or friends (*n* = 16, 36%). Some patients found dating difficult, or avoided it entirely, due to low confidence and fear of judgement or rejection due to their AA. Two patients described hiding their AA from romantic partners early in their relationship; however, the same patients later found these relationships to be a source of support for them in coping with AA. Patients generally described their family as supportive but noted that family members were initially shocked and lacked information about AA following their diagnosis. The impact of AA on friendships also varied; some patients cited friendship as a source of support but others had withdrawn from friendships, lost friends who had been unsupportive, or would only confide in very close friends.

-
  *“It’s made me like harder to talk to girls, fear of like being judged because I look different.” (22-M)*
-
  *“I’m not as outgoing as I was. I don’t want to go make new friends and meet new people. [ …] I’d rather just kind of stay home and be alone so I don’t have to deal with people asking and looking at me.” (27-F)*

## Discussion

This qualitative interview study provided insight into the effects of AA on patients’ lives. The first known conceptual model of the effects of AA was developed, which can provide an evidence base for identifying important treatment outcomes and for selecting and developing disease-specific PRO measures for future AA clinical research. Findings from this study also contribute to our understanding of the impact of AA on individuals, which can provide a foundation for identifying the additional support that people living with AA may require. This awareness is essential for healthcare professionals to identify and seek to address patients’ unmet needs.

The psychosocial effects of AA are just as important -- if not more important -- than the physical effects of AA, with many patients describing feelings of grief, helplessness, and depression, and reporting considerable impact on their lifestyles and relationships. The psychosocial effects of AA were related to its physical effects. Hair loss in areas that were visible to and could therefore be judged by others were the primary cause of patients’ psychosocial insecurities. For most patients, scalp hair loss, firstly, and eyebrow loss, secondly, were the most bothersome physical effects of AA. Patients with AA are living with a condition with no remission-sustaining treatments that for many has caused nearly a lifetime of psychosocial struggles.

These findings reflect the results of previous qualitative studies, which have also found AA to be emotionally devastating and to have considerable impact on self-esteem and social confidence [18, 20, 22, 23]. Additionally, our qualitative findings further conceptualize the findings of quantitative studies described in a recent systematic review that identified reduced HRQoL, anxiety and depression as key psychosocial comorbidities of patients with AA [7–15, 36].

The postulation that females may be more emotionally affected than males [19–21] was not reflected in these interviews; males appeared to be as likely as females to describe substantial psychosocial effects of AA. Males expressed insecurities about their appearance, unease in social situations, and feared judgement and rejection from others. Our findings do suggest, however, that there may be gender differences in the causes of the emotional distress experienced by patients with AA; eyebrow loss was named as the most bothersome sign/symptom of AA exclusively by male patients, perhaps because: 1) the cosmetic concealment of eyebrow loss (e.g. with make-up or microblading) is more socially acceptable for females, and 2) it is generally acceptable for males to have no scalp hair (all hair missing or a shaved head). Although other studies have reported the psychosocial impacts of eyebrow loss [18, 22], further exploration of this topic is warranted.

While findings from these interviews support a previous study that reported wearing a wig has a positive impact on quality of life [17], it was also identified that concealment of AA did not fully alleviate patients’ insecurities or distress. Indeed, our study found that wearing a wig or cosmetics (e.g. to conceal eyebrow or eyelash loss) is associated with other unique psychosocial impacts and can instigate considerable anxiety due to fears of displacement. This anxiety, just as the anxiety caused by unconcealed differences in appearance, led to social avoidance that could impair relationships, limit lifestyles, and further increase feelings of isolation and depression [22].

Existing disease-specific quality of life measures for AA have been developed with little or no patient involvement [37–39] and do not appear to be widely used [17]. Although the Skindex [40–42] is not disease-specific and was also developed with limited qualitative input from patients, it has a strong theoretical framework [25, 26] and has been used in AA [8, 15]. The concepts that emerged in this study broadly corresponded with physical and psychosocial domains of the Skindex frameworks of the burden of skin disease [25, 26], although further subdomains were added to capture the full impact of AA found in this study. The conceptual framework and conceptual model of the effects of AA that we developed suggested that the Skindex may be an appropriate conceptual assessment measure of the psychosocal impact of AA. However, each item within the Skindex asks patients about ‘your skin condition’ and our work to adapt the Skindex-16 [42] to be appropriate for AA/hair loss (e.g. revising items to ‘your alopecia’, etc.) is ongoing and includes testing with patients. In addition, a comprehensive suite of content-valid clinical outcome assessments of the key physical effects of AA were developed as part of this study. Development of an investigator global assessment [43] and PRO measure [27] for scalp hair loss, along with ClinRO and PRO measures for eyebrow loss, eyelash loss, nail appearance and eye irritation [28] are reported elsewhere.

This study included 45 patients from North America (*n* = 42 US, *n* = 3 Canada) who had experienced severe or very severe AA (≥50% scalp hair loss). In addition, patients with experience of eyebrow and/or eyelash loss were over-sampled in this study to allow fuller exploration of the impacts associated with AA hair loss in areas other than the scalp. The psychosocial effects of milder AA were not investigated. The sample size was large for a qualitative study [34]. Data saturation (i.e. when no new relevant concepts emerge) was assessed in line with industry best practice [32, 33] and met in the first 24 interviews. Our findings provide rich data on the psychosocial experience of AA and are mostly consistent with results of other studies [18–23] as described above. Although quality of life impacts have been reported in patients with AA in other countries using non-disease-specific PRO measures [7, 9–13], the specific cultural implications of hair loss may differ globally and, therefore, qualitative exploration in other countries and cultures is warranted.

## Conclusion

The physical signs/symptoms of AA are associated with considerable emotional and functional impairment among both men and women. Patients go to great lengths to conceal their hair loss, including avoiding everyday activities that risk exposure. There is a need for additional disease awareness and support for individuals living with AA, and importantly, for remission-sustaining treatments because of these significant quality of life impacts.

## Data Availability

The datasets generated during and/or analysed during the current study are not publicly available due to confidentiality of the information. However, the corresponding author can be contacted for any data related questions.

## Abbreviations

AA: Alopecia Areata
CAD: Canadian Dollar
ClinRO: Clinician-reported Outcome
HRQoL: Health-related quality of life
ICF: Informed consent form
JAKi: Janus kinase inhibitors
PRO: Patient-Reported Outcome
SALT: Severity of Alopecia Tool
USD: United States Dollar
